# Clinical characteristics of meconium aspiration syndrome in neonates with different gestational ages and the risk factors for neurological injury and death: A 9-year cohort study

**DOI:** 10.3389/fped.2023.1110891

**Published:** 2023-03-07

**Authors:** Lei Luo, Meng Zhang, Jun Tang, Wenxing Li, Yang He, Yi Qu, Dezhi Mu

**Affiliations:** ^1^ Department of Pediatrics, West China Second Hospital, Sichuan University, Chengdu, China; ^2^ Key Laboratory of Birth Defects and Related Diseases of Women and Children, Sichuan University, Ministry of Education, Chengdu, China

**Keywords:** meconium aspiration syndrome, gestational age (GA), clinical characteristics, neurological injury, risk factors

## Abstract

**Background:**

The presence of meconium is associated with gestational age, and the incidence of meconium aspiration syndrome (MAS) increases with gestational age. Our study compared the differences in the clinical characteristics of patients with MAS at different gestational ages and discussed the risk factors for neurological injury and death from MAS.

**Methods:**

A total of 294 neonates diagnosed with MAS between 2013 and 2021 were included. Patients were divided into preterm, early-term, full-term, and late-term groups according to gestational age. We compared the patients’ basic demographic, treatment, complications, and clinical outcomes in the different groups. We also analyzed the risk factors of neurological injury and death in patients with MAS.

**Results:**

The mean age at admission (0.55 ± 0.9 h) was lower and the proportion of cesarean deliveries (90.00%, 27/30) was higher in the preterm group than in the other three groups. There was no statistically significant difference among the four groups regarding 1- and 5-min Apgar scores and the need for delivery room resuscitation. In terms of complications, early-term infants had the highest incidence of neurological injury (52.9%, 27/51), and late-term infants had the highest incidence of pneumothorax (37.8%, 17/45). The overall mortality rate of children with MAS was 7.80% (23/294), and the difference in mortality rates among the four groups was not significant. Low 1-min Apgar score and gestational age, metabolic acidosis, and respiratory failure were independent risk factors for neurological injury; metabolic acidosis, respiratory failure, and sepsis were independent risk factors for death in neonates with MAS.

**Conclusion:**

The clinical characteristics of MAS neonates of different gestational age are different mainly in complications. Early-term infants are more likely to complicate with neurological injury, and late-term infants are more likely to complicate with pneumothorax. Low 1-min Apgar score and gestational age, metabolic acidosis, and respiratory failure were established as risk factors for neurological injury; metabolic acidosis, respiratory failure, and sepsis were independent risk factors for death in neonates with MAS.

## Introduction

Meconium aspiration syndrome (MAS) is a syndrome in which the fetus inhales meconium-contaminated amniotic fluid during delivery or *in utero* (1), resulting in chemical inflammation, airway obstruction, and a series of systemic symptoms, with high morbidity and mortality rates (2). Severe MAS can be complicated by acute respiratory distress syndrome (ARDS), air leak syndrome, respiratory failure, persistent pulmonary hypertension (PPHN), and multiorgan functional impairment. PPHN has an important impact on severity and prognosis (3, 4).

During pregnancy, 4%–22% of women have meconium-stained amniotic fluid (MSAF) (5) and approximately 3%–12% of neonates with MSAF develop MAS (6). MSAF is rare before 34 weeks of gestation and occurs in only 2% of neonates with a gestational age <37 weeks. However, it affects 44% of neonates with a gestational age of over 42 weeks (7). The incidence of MSAF can be as high as 23%–52% when gestation is over 42 weeks (8). The mortality rate of neonates with MAS is up to 13.3% (9). The presence of meconium is related to gestational age, and the incidence of MAS increases with gestational age (10, 11), from 0.24 to 1.42 per 1,000 births between 38 and 42 weeks (12). The risk of MAS is approximately four times higher in neonates born at 42 weeks of gestation than in those born at 37 weeks. Moreover, the risk increases by 27 times when the gestational age reaches 43 weeks (5). This increase in the incidence of MAS with gestational age may be associated with the stimulation of gastrointestinal motility by vagal excitation when the fetus undergoes hypoxic stress or umbilical cord compression. Full- and post-term fetuses are more likely to expel meconium in these situations than preterm infants, and the maturity of the gastrointestinal tract in preterm infants may limit their meconium expulsion (13). Gastric motility and secretion increase with gestational age, facilitating the expulsion of meconium (14). Some studies suggest that the severity of MAS is related to the following factors, including low 5-minute Apgar score, fetal distress, fetal heart rate abnormalities, cesarean delivery and need of surfactant therapy (5, 15, 16), (33–36).

However, few studies have explored whether the clinical characteristics of patients with MAS change with gestational age. Also, few studies have discussed the risk factors for neurological injury and death in neonates with MAS. Therefore, our study retrospectively analyzed the clinical data of patients with MAS at our hospital over the past nine years. We compared the differences in the clinical characteristics of patients with MAS at different gestational ages and discussed the risk factors for neurological injury and death due to MAS.

## Materials and methods

### Patients and study design

Our study included 294 newborns with MAS admitted to the Second West China Hospital of Sichuan University between January 1, 2013 and December 31, 2021. MAS was defined as a clinical condition characterized by respiratory failure occurring in neonates born through MSAF whose symptoms cannot be otherwise explained and with typical radiological characteristics (8). The inclusion criteria were as follows: neonates with (1) MSAF, (2) respiratory symptoms, such as moaning, shortness of breath, and respiratory distress after birth, and (3) evidence of MAS on imaging examination. The exclusion criteria were as follows: (1) congenital (inherited) metabolic diseases or chromosomal disorders; (2) congenital developmental malformations of the heart, airway, and lungs; and (3) incomplete data. Newborns were divided by gestational age into preterm (gestational age <37 weeks), early-term (37 weeks ≤ gestational age <39 weeks), full-term (39 weeks ≤ gestational age <41 weeks), and late-term (gestational age ≥41 weeks, including post-term infants with gestational age ≥42 weeks) groups. Since there were only six cases of post-term infants, they were included in the late-term infant group.

### Clinical data collection

The clinical data of MAS neonates, including (1) basic demographic and clinical characteristics of neonates: gestational age, sex, age at admission, 1- and 5-min Apgar scores, mode of delivery, fetal distress (17), and delivery room resuscitation (including continuous positive pressure ventilation and tracheal intubation); (2) perinatal characteristics of mothers, including maternal age, gravidity and delivery, number of cesarean sections, premature rupture of membranes, umbilical cord abnormalities (mainly including umbilical cord around neck and umbilical cord torsion), and presence of diabetes, intrahepatic cholestasis, or hypothyroidism during pregnancy (the top three common maternal disease during pregnancy based on our data); (3) treatment: mode and duration of respiratory support treatment, use of pulmonary surfactant (PS), antibiotic use and duration, use of hypothermia treatment, and inhaled nitric oxide (iNO) (17); and (4) complications and regression: respiratory failure, neurological injury, metabolic acidosis, pneumothorax, respiratory acidosis, sepsis, ARDS, pulmonary hemorrhage, PPHN, and death were collected. Fetal distress was mainly diagnosed through fetal heart rate monitoring, including late deceleration, severe bradycardia, severe variable deceleration and disappearance of fetal heart rate baseline. The definition of respiratory failure was clinically and blood gas confirmed hypoxemia requiring intratracheal mechanical ventilation (MV) for at least 24 h (18). Neurological injuries mainly included intracranial hemorrhage, hypoxic-ischemic encephalopathy (HIE) or white matter injury in preterm infants, which was diagnosed through brain imaging or electrophysiological examination. Neonatal sepsis was diagnosed through clinical symptoms or in the presence of a positive blood culture, along with clinical and laboratorial parameters (white blood cell count, platelet count, reactive C protein) (19). Deaths were defined as in-hospital deaths or deaths within 24 h of discharge from the hospital.

### Statistical analysis

Kruskal–Wallis, chi-square, and Fisher's exact tests were used to examine group differences for each dependent variable. A *P* value < 0.05 was considered significant for comparison among the four groups. If significant, the Mann–Whitney U test and chi-square decomposition were applied to determine significant differences between various pairs of groups. Since multiple comparisons were performed on the same data, the significance level was adjusted using Bonferroni correction, with the accepted level of significance set at *P* < 0.008.

When analyzing the risk factors for neurological injury and death, the rank sum and chi-square tests were used for univariate analysis. Risk factors for univariate analysis of neurological injury included gestational age; sex; age at admission; 1- and 5-min Apgar scores; mode of delivery; fetal distress; maternal age; gravidity and delivery; number of cesarean sections; premature rupture of membranes; umbilical cord abnormalities; maternal diabetes, intrahepatic cholestasis, and hypothyroidism; maternal antibiotic use; respiratory failure; metabolic acidosis; pneumothorax; respiratory acidosis; sepsis; ARDS; pulmonary hemorrhage; and PPHN. The risk factors used for the univariate analysis of death included the abovementioned factors plus neurological injury. Factors with statistical differences in the univariate analysis were considered in the multivariate logistic regression analysis. Statistical analyses were performed using SPSS software (version 24.0; SPSS Inc., Chicago, IL, USA). Statistical significance was set at *P* < 0.05.

## Results

### Comparison of basic demographic and clinical characteristics

This study included 294 neonates with MAS. The mortality rate was 7.8%. The number of MAS cases, the proportion of MAS patients of inpatients in our NICU and the proportion of referral cases per year were showed in Table 1. The study included 166 (56.46%) male and 128 (43.54%) female infants. After grouping by gestational age, there were 30 (10.20%), 51 (17.35%), 168 (57.14%), and 45 (15.31%) infants in the preterm, early-term, full-term, and late-term infant groups, respectively; 140 (47.62%) of the infants were delivered in our hospital, and 154 (52.38%) were transferred from other hospitals. The gestational age in preterm infants group ranged from 31^2/7^ to 36^6/7^ weeks. There were no statistically significant differences (*P* > 0.05) among the four groups regarding sex, 1- and 5-min Apgar scores, and the need for delivery room resuscitation (Table 2). The mean age at admission was lower (0.55 ± 0.9 h) and the proportion of cesarean deliveries (90.00%, 27/30) was higher in the preterm group than in the other three groups, with statistically significant differences (*P* < 0.008). The incidence of intrauterine distress was higher in the preterm group than in the late-term group (*P* < 0.008, Supplementary Table S1).

**Table 1:** Cases of MAS in nine years [*n* (%)].

	2013	2014	2015	2016	2017	2018	2019	2020	2021
Inpatients in NICU	3,582	3,397	3,627	3,635	3,105	3,945	5,068	6,001	6,544
Cases with MAS^a^	54 (1.5)	48 (1.4)	38 (1.1)	28 (0.8)	22 (0.7)	20 (0.5)	32 (0.6)	25 (0.4)	27 (0.4)
Referral cases with MAS^b^	19 (35.2)	21 (43.8)	23 (60.5)	7 (25.0)	7 (31.8)	9 (45.0)	27 (84.4)	21 (84.0)	19 (70.4)
MAS-related death	5 (9.3)	2 (4.2)	5 (13.2)	4 (14.3)	1 (4.5)	0 (0.0)	4 (12.5)	1 (4.0)	1 (3.7)

^a^ Cases with MAS including neonates born in our hospital and referral neonates.

^b^ The proportion of referral cases = the number of referral cases / the number of total MAS cases.

**Table 2:** Comparison of basic demographic and clinical characteristics of patients with different gestational age.

[*n* (%) or median (IQR)]						
	Preterm group *n* = 30	Early term group *n* = 51	Full term group *n* = 168	Late term group *n* = 45	*P*	Total *n* = 294
Neonates						
Gestational age (weeks)	35.40(2.20)	38.10(1.00)	40.00(0.70)	41.10(0.40)		39.70(1.90)
Male	15 (50.0)	35 (68.6)	93 (55.4)	23 (51.1)	0.243	166 (56.5)
Age (hours)	0.55(0.9)	3.50(25.4)	2.39(12.19)	2.35(15.47)	**<0.001** *	2.20(14.50)
1-minute Apgar	7.0 (4.50)	7.00 (4.00)	8.00 (3.00)	8.00 (4.00)	0.490	8.00 (4.00)
5-minute Apgar	8.0 (3.00)	9.00 (2.00)	9.00 (3.00)	9.00 (2.25)	0.790	9.00 (3.00)
Cesarean delivery	27 (90.0)	31 (60.8)	81 (48.2)	24 (53.3)	**<0.001** *	163 (55.4)
Fetal distress	Diagnosed:17 (56.7)	Diagnosed: 26 (51.0)	Diagnosed: 54 (32.1)	Diagnosed: 13 (28.9)	**0.011** *	Diagnosed: 110 (37.4)
	Suspicious:0 (0.0)	Suspicious: 5 (9.8)	Suspicious: 22 (13.1)	Suspicious: 9 (20.0)		Suspicious: 36 (12.2)
Delivery room resuscitation	24 (80.0)	29 (56.9)	98 (58.3)	26 (57.8)	0.141	177 (60.2)
Mothers						
Maternal age (years)	31.50(7.75)	29.50(7.25)	29.00(5.25)	29.00(6.00)	0.130	29.00(6.50)
Times of gravidity	2.00 (3.00)	2.00 (1.00)	2.00 (1.00)	1.00 (1.00)	**0.040** *	2.00 (1.00)
Times of delivery	2.00 (1.00)	1.00 (1.00)	1.00 (0.00)	1.00 (0.00)	**<0.001** *	1.00 (1.00)
Times of cesarean sections	1.00 (1.00)	1.00 (1.00)	0.00 (1.00)	1.00 (0.00)	**<0.001** *	1.00 (1.00)
Premature rupture of membranes	9 (30.0)	11 (21.6)	29 (17.3)	8 (17.80)	0.412	57 (19.4)
Umbilical cord abnormalities	14 (46.7)	23 (45.1)	69 (41.1)	18 (40.0)	0.897	124 (42.2)
Gestational diabetes	10 (33.3)	8 (15.7)	31 (18.5)	1 (2.2)	**0.005** *	50 (17.0)
Intrahepatic cholestasis of pregnancy	11 (36.7)	7 (13.7)	9 (5.4)	0 (0.0)	**<0.001** *	27 (9.2)
Hypothyroidism of pregnancy	4 (13.3)	4 (7.8)	20 (11.9)	2 (4.4)	0.430	30 (10.2)

* *P *< 0.05.

The mean age of the mothers was 29.16 (19–41) years, with no statistical difference among the four groups. The mothers' gravidity was higher in the preterm group than in the late-term group; the number of deliveries was higher in the preterm group than in the full-term and late-term groups, and the number of previous cesarean deliveries was higher in the preterm group than in the other three groups. All differences were statistically significant (*P* < 0.008) (Supplementary Table S1). There was no significant difference in the incidence of premature rupture of membranes and umbilical cord abnormalities among the four groups. Regarding maternal complications during pregnancy, the incidence of maternal gestational diabetes was significantly higher in the preterm group (33.3%, 10/30) than in the late-term group (2.20%, 1/45); the incidence of intrahepatic cholestasis during pregnancy was higher in the preterm group (36.70%, 11/33) than in the full-term (5.40%, 9/168) and late-term groups (0.00%, 0/45; *P* < 0.008). There was no significant difference in the incidence of gestational hypothyroidism among the four groups. In our study, the maternal antibiotic was used when premature rupture of membranes or infection (maternal fever, or positive cervical secretions cultures for pathogens) were considered, according to the perinatal antibiotic use practices of the Department of Obstetrics in our hospital. Perinatal infection is common in China. Therefore, in clinical practice, obstetricians sometimes conduct cervical secretion culture for high-risk pregnant women. Sixty-two mothers (21.09%, 62/294) used antibiotics during the prenatal period.

### Comparison of treatment methods

In total, 158 (53.70%, 158/294) patients in this study were treated with mechanical ventilation (including noninvasive or invasive mechanical ventilation); 264 (89.80%) were treated with antibiotics, and the average duration of antibiotic treatment was 168.25 h. Twenty-eight (9.52%) infants were diagnosed with PPHN, and 23 (82.14%) of these were treated with iNO, accounting for 7.80% (23/294) of the overall patients with MAS. A total of 27 (9.18%) patients with MAS were treated with hypothermia, with the highest usage proportion observed in the full-term group (11.90%, 20/168). One patient in the early-term group who was treated with extracorporeal membrane oxygenation (ECMO), survived and was discharged (Table 3).

**Table 3:** Comparison of treatment of patients with different gestational age [*n* (%) or median (IQR)].

	Preterm group *n* = 30	Early term group *n* = 51	Full term group *n* = 168	Late term group *n* = 45	*P*	Total *n* = 294
Mechanical ventilation	16 (53.3)	30 (58.8)	90 (53.6)	22 (48.9)	0.805	158 (53.7)
Non-invasive only	1 (3.3)	11 (21.6)	19 (11.3)	2 (4.4)	**0.032** *	33 (11.2)
Invasive and non-invasive	15 (50.0)	19 (37.3)	71 (42.3)	20 (44.4)	0.724	125 (42.5)
Pulmonary surfactant	11 (36.7)	13 (25.5)	38 (22.6)	11 (24.4)	0.447	73 (24.8)
Antibiotics	26 (86.7)	48 (94.1)	152 (90.5)	38 (84.4)	0.399	264 (89.8)
Duration of antibiotic therapy (h)	173.15(156.82)	181.30(187.00)	166.50(152.40)	165.80(275.36)	0.930	168.25(162.50)
Hypothermia therapy	1 (3.3)	3 (5.9)	20 (11.9)	3 (6.7)	0.376	27 (9.2)
Inhaled nitric oxide	1 (3.3)	6 (11.8)	11 (6.5)	5 (11.1)	0.402	23 (7.8)
ECMO	0 (0.0)	1 (2.0)	0 (0.0)	0 (0.0)	0.429	1 (0.3)
Duration of hospitalization (days)	11.00(6.38)	9.00(6.90)	8.00(7.00)	10.00(9.50)	0.510	9.00(7.00)

* *P *< 0.05.

### Comparison of complications and regression

The rate of positive sputum cultures for pathogens was 10.88% (32/294). The top three pathogens were coagulase-negative *Staphylococcus* (4.08%, 12/294), *Escherichia coli* (2.38%, 7/294), and *Streptococcus gramineus* (1.36%, 4/294). There was no significant difference in the positivity rates among the groups. Two hundred and forty-six children completed the blood culture examination, and 1.22% (3/246) of them were positive, including one infant (*Listeria monocytogenes*) in the preterm group and two (one case of *Listeria monocytogenes* and another of *Staphylococcus epidermidis*) in the full-term group. Fifty-three patients completed cerebrospinal fluid culture; nine of them (16.98%) were in the preterm group, seven (13.21%) in the early-term group, 26 (49.06%) in the full-term group, and 11 (20.75%) in the late-term group. Cerebrospinal fluid cultures were positive (for *Enterococcus faecalis*) in only one infant in the full-term group.

The complications included respiratory failure (41.80%, 123/294), neurological injury (36.10%, 106/294), metabolic acidosis (31.00%, 91/294), pneumothorax (18.40%, 54/294), respiratory acidosis (16.00%, 47/294), sepsis (15.60%, 46/294), ARDS (15.30%, 45/294), pulmonary hemorrhage (10.50%, 31/294), and PPHN (9.50%, 28/294). The top three complications were respiratory failure, neurological impairment, and metabolic acidosis. There was no statistical difference in the incidence of respiratory failure, metabolic acidosis, respiratory acidosis, sepsis, ARDS, pulmonary hemorrhage, or PPHN among the four groups (*P* > 0.05, Table 4). Early-term infants had the highest incidence of combined neurological injury (52.9%, 27/51); late-term infants had the highest incidence of pneumothorax (37.8%, 17/45). The incidence of pneumothorax was higher in late-term infants than in full-term infants (13.1%, 22/168), and the incidence of neurological injury was higher in early-term infants than in late-term infants (24.4%, 11/45), with a statistically significant difference (*P* < 0.008, Supplementary Table S2).

**Table 4:** Comparison of complications and regression of patients with different gestational age [*n* (%)].

	Preterm group *n* = 30	Early term group *n* = 51	Full term group *n* = 168	Late term group *n* = 45	*P*	Total *n* = 294
Positive sputum culture	2 (6.7)	4 (7.8)	18 (10.7)	8 (17.8)	0.407	32 (10.88)
Respiratory failure	14 (46.7)	18 (35.3)	68 (40.5)	23 (51.1)	0.413	123 (41.8)
Neurological injury	14 (46.7)	27 (52.9)	54 (32.1)	11 (24.4)	**0.009** *	106 (36.1)
Metabolic acidosis	5 (16.7)	18 (35.3)	52 (31.0)	16 (35.6)	0.290	91 (31.0)
Pneumothorax	6 (20.0)	9 (17.6)	22 (13.1)	17 (37.8)	**0.002** *	54 (18.4)
Respiratory acidosis	7 (23.3)	9 (17.6)	25 (14.9)	6 (13.3)	0.624	47 (16.0)
Sepsis	8 (26.7)	8 (15.7)	22 (13.1)	8 (17.8)	0.276	46 (15.6)
ARDS	7 (23.3)	7 (13.7)	27 (16.1)	4 (8.9)	0.389	45 (15.3)
Pulmonary hemorrhage	1 (3.3)	5 (9.8)	21 (12.5)	4 (8.9)	0.547	31 (10.5)
PPHN	1 (3.3)	7 (13.7)	15 (8.9)	5 (11.1)	0.461	28 (9.5)
Death	3 (10.0)	5 (9.8)	11 (6.5)	4 (8.9)	0.701	23 (7.8)

* *P *< 0.05.

The overall mortality rate of patients with MAS was 7.80% (23/294), and the mean age of the patients was 52.18 ± 48.56 h. The mortality rates in the four groups at different gestational ages were 10.00% (3/30), 9.80% (5/51), 6.50% (11/168), and 8.90% (4/45), respectively, with no significant differences (*P* > 0.05, Table 4).

### Risk factors for neurological injury and death in patients with MAS

Univariate analysis revealed that the following risk factors: premature rupture of membranes: metabolic acidosis, respiratory acidosis, respiratory failure, ARDS, pulmonary hemorrhage, PPNH, sepsis, low gestational age, early age at admission, and low 1- and 5-min Apgar scores. Multivariate logistic regression analysis using these variables as independent variables showed that a low 1-min Apgar score (OR 0.77, *P* < 0.001), low gestational age (OR 0.82, *P* = 0.022), metabolic acidosis (OR 2.26, *P* = 0.007), and respiratory failure (OR 3.02, *P* < 0.001) were independent risk factors for neurological injury in patients with MAS (Table 5).

**Table 5:** Multivariable logistic analysis of risk factors of neurological injury in patients with MAS.

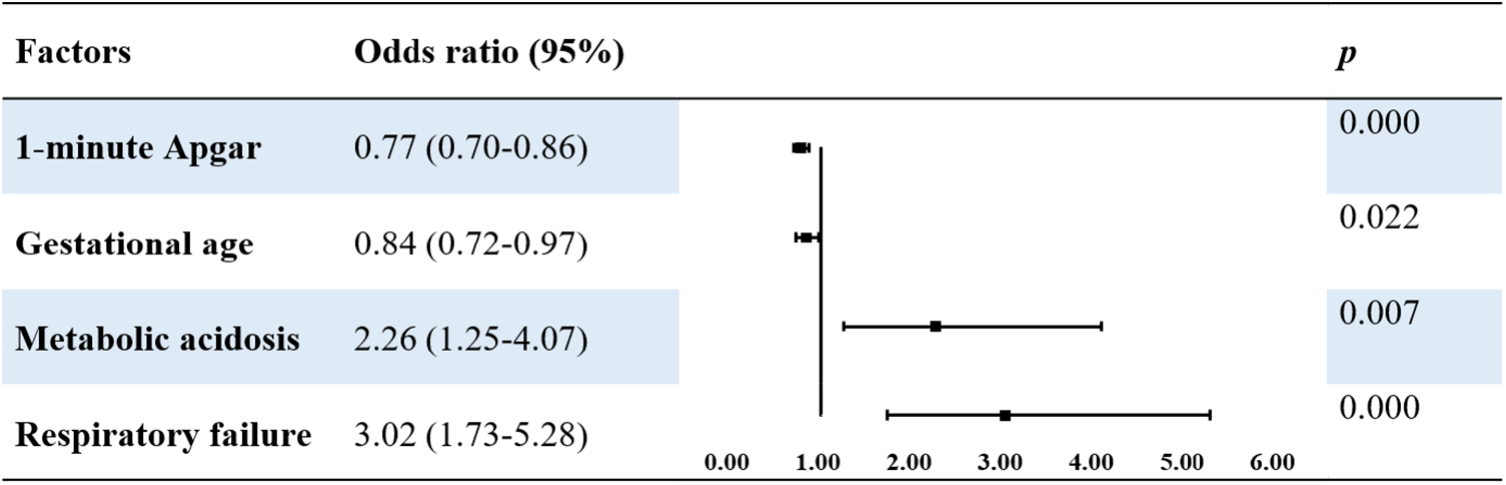

According to the univariate analysis, the following risk factors increased the risk of death in patients with MAS: fetal intrauterine distress, metabolic acidosis, respiratory acidosis, respiratory failure, ARDS, pulmonary hemorrhage, PPNH, and sepsis (*P* < 0.05). Multivariate logistic regression analysis using these risk factors as independent variables showed that respiratory failure (OR 3.91, *P* < 0.001), sepsis (OR 5.49, *P* = 0.010), and metabolic acidosis (OR 7.04, *P* = 0.010) were independent risk factors for death in children with MAS (Table 6).

**Table 6:** Multivariable logistic analysis of risk factors of death in patients with MAS

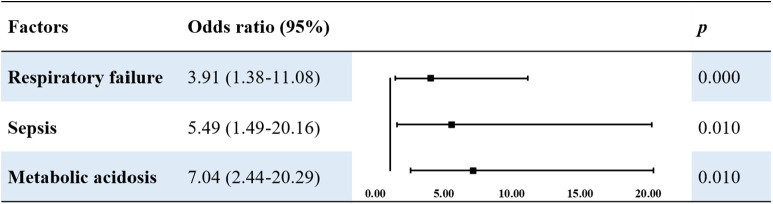

## Discussion

Meconium begins to gradually form in the fetal gastrointestinal tract at 14–16 weeks of gestational age and is usually excreted within 24 h of birth (20). However, when the fetus experiences stressful conditions, such as acidosis and hypoxia, it causes a vagal response that triggers increased peristalsis of the gastrointestinal tract and relaxation of the anal sphincter, leading to the entry of the meconium into the uterine cavity and causing MSAF (8). Neonates born to mothers with MSAF are more likely to develop complications such as neonatal sepsis, seizures, and neurological damage (21). Inhalation of MSAF *in utero* or after birth in neonates results in the development of MAS, which occurs through pathophysiological mechanisms, including inflammation and inflammatory cascade reactions, mechanical obstruction of the airway, and PS inactivation (2). MAS is a common critical neonatal illness with complex etiologies, including fetal distress, unstable fetal heart rate, emergency cesarean delivery, low Apgar score, and intrauterine fetal growth retardation (22).

MAS accounts for 10% of neonatal respiratory failures, and the mortality rate could be as high as 20% in developing countries (23). The incidence of MAS in China is approximately 1.2–2.2%, and the mortality rate is approximately 7.0–15.0% (24). The incidence of neonatal MAS in Nepal is approximately 6.6–8.6%, with a mortality rate of 11.3% (14). According to data from a large cohort in Australia and New Zealand, the mortality rate of MAS in developed countries was 2.5% (25). In our study, the mortality rate of MAS in 2021 was decreased to 3.7%.The incidence of MAS has decreased in recent years, which may be attributed to elective induction of labor in pregnancies beyond 41 weeks, aggressive management of fetal distress, and birth asphyxia (26); however, the severity of MAS has not decreased (27).

Our study showed that differences in perinatal characteristics among the four groups were mainly found between the preterm group and the other groups. Although MAS was rare in neonates with gestational age less than 34 weeks, we still found patients less than 34 weeks with the evidence of MAS in our study. MAS mostly occurred in term infants, so the number of patients in premature group was the least. The incidence rates of intrauterine distress (56.7%, 17/30) and cesarean delivery (90.00%, 27/30) were higher in the preterm group than in the other three groups, which may be related to the adverse history of delivery and multiple maternal comorbidities in preterm mothers. The mothers of infants in the preterm group had a significantly higher incidence rate of previous cesarean deliveries, diabetes, and intrahepatic cholestasis during pregnancy than those of infants in the other three groups.

The current treatment for MAS is supportive and includes oxygen therapy, mechanical ventilation, and intravenous rehydration. In recent years, the use of PS, iNO, high-frequency mechanical ventilation, and ECMO has made saving more patients with MAS possible (28). In this study, 28 (9.52%) patients received combined PPHN, and 23 were treated with iNO (82.14%, 23/28). iNO affects vascular smooth muscle, causing pulmonary vasodilation in the region of pulmonary ventilation, and reduces ventilation-perfusion mismatch to improve oxygenation (29). iNO therapy reduces ECMO requirements and mortality in patients with hypoxic respiratory failure and PPHN (28). Patients with severe MAS and respiratory failure can be treated with ECMO. Studies have shown that up to 35% of infants require ECMO due to MAS and that the survival rate of patients with MAS can exceed 94% after treatment with ECMO (30). ECMO in neonates was only recently introduced in our center; therefore, only one child with MAS was successfully treated with ECMO in this study. The use of ECMO is limited in developing countries; therefore, our data may provide a reference for regions where ECMO is not yet available. Antibiotics were used in 89.8% (264/294) of the patients in our study, which was similar to the study from the USA (92%, 6906/7518) (23). In clinical practice, it is difficult to completely eliminate intrauterine infection at the beginning of MAS, and secondary infection may occur later, so the proportion of antibiotics use was usually high.

A study found that antibiotic use in patients with MAS did not significantly reduce the risk of mortality, length of hospital stay, or incidence of sepsis (31). Kelly et al. found that antibiotics did not reduce the risk of sepsis in patients with MAS (32). Therefore, more clinical evidence is needed to determine the effectiveness of antibiotics in MAS treatment.

Symptoms in patients with MAS range from mild shortness of breath to severe respiratory distress and various serious complications, such as pulmonary hypertension, sepsis, pulmonary hemorrhage, and neurological damage. In this study, respiratory failure, neurological injury, and metabolic acidosis were the top three complications. The differences in the incidence of neurological injury among patients with different gestational ages were statistically significant. Early-term infants had the highest incidence rate of neurological injury, and the difference was statistically significant compared with that in late-term infants. Preterm and early-term infants were more likely to have neurological injuries such as intracranial hemorrhage. Pneumothorax is an important indicator of MAS severity. In a cohort study including 1,061 patients with MAS, pneumothorax was present in 42% of the patients who died (25). The incidence of pneumothorax in our study was 18.37% (54/294), and 19.37% (4/23) of the patients who died of MAS had a pneumothorax. The difference in the incidence of pneumothorax between patients of different gestational ages was statistically significant, and the incidence of pneumothorax was highest in the late-term group.

This study also investigated independent risk factors for neurological injury and death in patients with MAS. We found that the risk factors for neurological injury included low 1-min Apgar scores, low gestational age, metabolic acidosis, and respiratory failure. MAS is a complex disease associated with neurological injury (33), and HIE reportedly increases mortality rate in patients with MAS (14, 34). Neonates with a lower gestational age have less tolerance for hypoxia and are more likely to develop neurological injuries. Hypoxemia is often observed in patients with MAS. The low 1-minute Apgar score observed in them suggests severe perinatal hypoxemia with secondary respiratory depression and metabolic acidosis, leading to pulmonary vasoconstriction and reduced pulmonary blood flow, which aggravate hypoxemia and metabolic acidosis, creating a spiral effect of pulmonary hypertension in MAS (7). Optimizing the timing of delivery could improve neurological outcomes. Blood gas analysis in this study showed the first results within 2 h after birth.

This study found that respiratory failure, metabolic acidosis, and sepsis were risk factors for mortality in patients with MAS. Respiratory failure is an independent risk factor for death and neurological injury. Correction of respiratory failure should be considered during treatment. Optimizing respiratory support methods, early use of PS and iNO, timely drainage of tension pneumothorax, and use of ECMO for those who meet these guidelines are essential. In this study, 15.6% (46/294) of patients with sepsis were diagnosed, and the mortality rate was 21.7% (10/46). Two children who died of sepsis had positive blood cultures for *Listeria*. Although *Listeria* infection is relatively rare in neonates, it can cause serious complications, such as central nervous system infection, acute respiratory distress syndrome, and death. Therefore, attention should be paid to bacterial infections in the perinatal period (35, 36).

Although many studies have shown that the incidence of MAS increases with gestational age, few have discussed the differences in the clinical characteristics of patients with MAS at different gestational ages. This study retrospectively analyzed the clinical data of 294 children with MAS over nine years to compare the similarities and differences in clinical characteristics among different gestational ages and identify the risk factors for neurological injury and death in patients with MAS; this study could serve as a reference for future diagnosis and treatment of patients with MAS at different gestational ages.

This study has some limitations. First, only single-center data from our hospital were included, and the number of patients in some groups was low; therefore, multicenter studies with larger sample sizes are required in the future. Second, this study only analyzed the clinical characteristics of the patients during hospitalization; the patients could have been followed up after discharge to monitor their prognosis. Moreover, because of the potential for poor outcome, the best approach for managing MAS is prevention. In this study, we only focus on the clinical characteristics of neonates with MAS, while in the future study, we will pay more attention to the risk factors of MAS and the treatment methods such as labor induction in reducing the risk of MAS.

In conclusion, the incidence of MAS has decreased in recent years, but the mortality rates fluctuate. There was no significant difference in treatment among neonates with MAS at different gestational ages. The incidence rate of pneumothorax was highest in late-term infants and that of neurological injury was highest in early-term infants. Low 1-min Apgar score, low gestational age, metabolic acidosis, and respiratory failure were independent risk factors for neurological injury in patients with MAS. Respiratory failure, metabolic acidosis, and sepsis were independent risk factors for death in children with MAS. Neonates with MAS should be closely monitored, adequately examined, and given timely supportive treatment. Moreover, patients with risk factors should be managed promptly to reduce the incidence of neurological injuries and mortality.

## Data Availability

The raw data supporting the conclusions of this article will be made available by the authors, without undue reservation.
