# Supplementary material for: Clinical characteristics of meconium aspiration syndrome in neonates with different gestational ages and the risk factors for neurological injury and death: A 9-year cohort study
Source: Front Pediatr. 2023 Mar 7;11:1110891. doi: 10.3389/fped.2023.1110891 (PMC10027737; doi:10.3389/fped.2023.1110891)
Supplement: Supplementary file 1 [file Table1.docx]

**Table s1** **P value of multiple comparison between basic demographic and clinical characteristics of patients with different gestational age**

|  | Preterm vs.  early term group | | Preterm vs.  full term group | Preterm vs.  late term group | Early term vs.  full term group | Early term vs.  late term group | Full term vs.  late term group |
| --- | --- | --- | --- | --- | --- | --- | --- |
| Age (h) | | **<0.001*** | **<0.001*** | **0.001*** | 0.298 | 0.474 | 0.871 |
| Fetal distress | | 0.276 | 0.009 | **0.004*** | 0.050 | 0.068 | 0.526 |
| Cesarean delivery | | **0.005*** | **<0.001*** | **0.001*** | 0.150 | 0.537 | 0.616 |
| Times of gravidity | | 0.303 | 0.095 | **0.007*** | 0.763 | 0.062 | 0.041 |
| Times of delivery | | 0.036 | **0.001*** | **<0.001*** | 0.405 | 0.014 | 0.032 |
| Times of cesarean sections | | **0.001*** | **<0.001*** | **<0.001*** | 0.330 | 0.506 | 0.856 |
| Gestational diabetes | | 0.096 | 0.085 | **<0.001*** | 0.685 | 0.034 | 0.008 |
| Intrahepatic cholestasis of pregnancy | | 0.026 | **<0.001*** | **<0.001*** | 0.062 | 0.014 | 0.209 |

****P*<0.008**

**Table s2** **P value of multiple comparison between complications of patients with different gestational age**

|  | Preterm vs.  early term group | Preterm vs.  full term group | Preterm vs.  late term group | Early term vs.  full term group | Early term vs.  late term group | Full term vs.  late term group |
| --- | --- | --- | --- | --- | --- | --- |
| Neurological injury | 0.649 | 0.145 | 0.079 | 0.008 | **0.006*** | 0.366 |
| Pneumothorax | 1 | 0.391 | 0.129 | 0.491 | 0.038 | **<0.001*** |

****P*<0.008**
